# TCA Cycle Rewiring as Emerging Metabolic Signature of Hepatocellular Carcinoma

**DOI:** 10.3390/cancers12010068

**Published:** 2019-12-25

**Authors:** Simona Todisco, Paolo Convertini, Vito Iacobazzi, Vittoria Infantino

**Affiliations:** 1 Department of Science, University of Basilicata, viale dell’Ateneo Lucano 10, 85100 Potenza, Italy; simona.todisco@unibas.it (S.T.); paolo.convertini@gmail.com (P.C.); 2 Department of Biosciences, Biotechnologies and Biopharmaceutics, University of Bari, via Orabona 4, 70125 Bari, Italy; vito.iacobazzi@uniba.it

**Keywords:** tricarboxylic acid (TCA) cycle rewiring, metabolic reprogramming, hepatocellular carcinoma (HCC), malate/aspartate shuttle (MAS), citrate/pyruvate shuttle, glutamine, NF-κB, HIF1

## Abstract

Hepatocellular carcinoma (HCC) is a common malignancy. Despite progress in treatment, HCC is still one of the most lethal cancers. Therefore, deepening molecular mechanisms underlying HCC pathogenesis and development is required to uncover new therapeutic strategies. Metabolic reprogramming is emerging as a critical player in promoting tumor survival and proliferation to sustain increased metabolic needs of cancer cells. Among the metabolic pathways, the tricarboxylic acid (TCA) cycle is a primary route for bioenergetic, biosynthetic, and redox balance requirements of cells. In recent years, a large amount of evidence has highlighted the relevance of the TCA cycle rewiring in a variety of cancers. Indeed, aberrant gene expression of several key enzymes and changes in levels of critical metabolites have been observed in many solid human tumors. In this review, we summarize the role of the TCA cycle rewiring in HCC by reporting gene expression and activity dysregulation of enzymes relating not only to the TCA cycle but also to glutamine metabolism, malate/aspartate, and citrate/pyruvate shuttles. Regarding the transcriptional regulation, we focus on the link between NF-κB-HIF1 transcriptional factors and TCA cycle reprogramming. Finally, the potential of metabolic targets for new HCC treatments has been explored.

## 1. Introduction

Hepatocellular carcinoma (HCC) is the most common primary liver cancer and the third leading cause of cancer-linked mortality, with an annual death rate exceeding 600,000 worldwide [1]. Viral infections (Hepatitis B and hepatitis C Viruses), toxins and drugs (e.g., alcohol and aflatoxin B1), non-alcoholic fatty liver disease, metabolic liver diseases, and diabetes are associated with HCC occurrence [2]. Despite progress in therapy and advanced screening of high-risk patients, many factors including the lack of effective therapeutic options for the advanced stages of the disease, the occurrence in the setting of liver disease, the aggressive and heterogeneous nature are responsible for the high mortality of HCC [3,4]. Thus, understanding molecular mechanisms underlying HCC pathogenesis and development is essential for innovative therapeutic interventions. Over recent years, increasing evidence has highlighted altered metabolic pathways as widespread as other cancer-associated features currently accepted as hallmarks of cancer [5]. Therefore, in addition to the classical hallmarks of cancer, the significance of some metabolites and/or key metabolic proteins have been taken into consideration for early diagnosis and better understanding of molecular mechanism of HCC.

Metabolic reprogramming is an essential hallmark of cancer, including HCC, as metabolic shifts represent a selective advantage for tumor growth, proliferation, and survival responding to the increased energy production, synthesis of macromolecules, and maintenance of redox balance. A well-known metabolic alteration in cancer cells is the elevated aerobic glycolysis, commonly referred as Warburg effect, which supports tumor growth [6]. However, only more recently the meaning and implications of this Warburg effect in cancer biology and its potential in diagnosis and drug targeting have begun to emerge. A markedly increased uptake of glucose in cancer, when compared to non-proliferating normal tissues, has been confirmed in a variety of tumors and positron emission tomography (PET) based on ^18^F-fluorodeoxyglucose (^18^F-FDG) uptake is a widespread diagnostic tool in oncology, as well as in monitoring the treatment effectiveness [7]. Excessive glycolytic flux—if not employed for biosynthesis—diverts to lactate allowing NAD^+^ cytosolic production and avoiding both glycolysis slowdown and TCA cycle suppression by an immoderate mitochondrial supply of NADH [8].

Warburg misinterpreted the meaning of aerobic glycolysis attributing it to a damaged mitochondrial respiration, which drives cancer cells to rely on alternative ways for energy production [9]. Today it is well-known that during cancer pathogenesis and development, a rewiring of metabolic pathways rather than a simple dysfunction is occurring in mitochondria [10]. They are reprogrammed to supply building blocks for nucleic acids, lipids, and protein synthesis; therefore, being essential for cancer cell proliferation. Accordingly, the Warburg effect is not an adaptive condition but a tightly regulated metabolic state supporting an increased biosynthetic demand.

Together with glucose, glutamine also supports cancer cell growth providing carbon as well as reduced nitrogen for de novo synthesis of nitrogen-containing compounds such as nucleotides, glucosamine-6-phosphate, and non-essential amino acids. It has been reported that cultured cancer cells may require up to 100-fold molar excess of glutamine compared to other amino acids [11] and glutamine deprivation from the tumor environment has been found in a variety of tumorigenic contexts [12,13]. As matter of fact, ^18^F-labeled glutamine tracer seems to be promising especially for tumors developing in areas such as brain where a weighty use of glucose occurs [14]. By glutaminase (GLS) and then glutamate dehydrogenase (GDH) or amino acid transaminase reactions, glutamine can be converted into α-ketoglutarate (αKG) thus representing the major anaplerotic substrate to replenish TCA cycle intermediates in proliferating cells [15]. Of note, glutamine-derived αKG may also provide citrate by a reductive carboxylation [16].

In this metabolic rewiring, the movement of metabolites across the mitochondrial membrane might play a crucial role for tumor growth. For example, citrate—the first intermediate of TCA cycle—is exported from mitochondria to the cytosol through the mitochondrial citrate carrier (CIC) resulting vital for de novo biosynthesis of fatty acids dramatically raised in cancer cells [17]. An increased lipogenesis also produces saturated lipids less susceptible to lipid peroxidation as adaptation to oxidative stress [18]. Indeed, CIC transport inhibition blocks tumor growth and its activity is increased in HCC [19,20]. Enzymes of de novo fatty acid synthesis including ATP-citrate lyase (ACLY), acetyl-CoA carboxylase, and fatty acid synthase are also overexpressed in a wide variety of solid human tumors, including HCC [21]. Of note, citrate export represents a further protection of mitochondria from excessive glycolysis because any pyruvate entering TCA cycle is channeled into this way.

In addition to citrate, TCA cycle supplies other metabolic precursors including aspartate and succinate. Export of aspartate from mitochondria is essential for protein, purine, and pyrimidine biosynthesis in proliferating tumor cells. Aspartate is exported to the cytosol by the mitochondrial aspartate/glutamate carriers (AGC1 and AGC2) in exchange for glutamate and a proton [22]. This transport is part of the malate/aspartate shuttle (MAS) whose activity is crucial for the regeneration of cytosolic NAD^+^ required for glycolysis. Very recently, cytosolic aspartate/glutamate carrier-derived aspartate has been described as an endogenous metabolic limitation for tumor growth, pointing out its role in cancer [23].

Succinate may function as a direct signaling messenger linking TCA cycle rewiring to tumorigenesis. In cancer cells, such as in innate immune cells [24], succinate moves outside the mitochondria via the dicarboxylic acid carrier and the voltage-dependent anion channel in the mitochondrial inner and outer membranes, respectively. Once in the cytosol, succinate inhibits some α-ketoglutarate-dependent oxygenases, including oxygen-dependent prolyl hydroxylase (PHD) enzyme [25] that hydroxylates the Hypoxia Inducible Factor 1α-subunit (HIF-1α) with subsequent proteosomal degradation. There is plenty of evidence that down-regulation as well as mutations of SDH genes, found in a variety of cancers, result in succinate accumulation, which inhibits HIF1-PHD, leading to HIF-stabilization and activation [26].

Thus, TCA cycle rewiring plays a central role in cancer cells being closely linked to the traffic of molecules between mitochondria and cytosol. Probably, its role in cancer metabolism, tumorigenesis, and cancer cell proliferation has been overlooked for a long time. In more recent years, in-depth analysis of cancer metabolism alterations has shown new roles for metabolic pathways, including TCA cycle, in tumors.

Considerably, this metabolic reprogramming results from altered gene expression of proteins involved in such pathways. Transcriptional regulation by transcriptional factors is responsible, at least in part, for these changes. Due to the growing evidence about role of the nuclear factor κB (NF-κB) in inflammation-linked cancers and oncogenesis [27] and the emerging interplay between HIF1 and TCA cycle, we have chosen to focus on these two factors, taking into account that they are not the only factors linked to altered energy metabolism in cancer. Therefore, in this review we look at gene expression and activity of TCA cycle and related enzymes such as citrate/piruvate and malate/aspartate shuttles and their potential role in HCC.

## 2. Inflammation and Role of NF-κB

Molecular mechanisms linking chronic inflammation and cancer have been suggested in initiation, promotion, and progression of tumors [28]. Indeed, macrophages are recruited in a variety of tumors [29]. Inflammatory process may foster multiple hallmarks of tumors such as growth factors sustaining proliferation and survival factors limiting apoptosis [5]. Moreover, inflammation can be evident at the earliest stages of neoplastic transformation and can promote the development of incipient neoplasias [29]. Inflammatory cells, largely macrophages, release chemicals including reactive oxygen species (ROS), which are mutagenic for surrounding cancer cells, accelerating their development [30]. Subsequently, as the tumors progress toward malignancy, the macrophage phenotype changes from the “inflammatory” (M1) to the “trophic” (M2) phenotype promoting angiogenesis and tissue formation [31].

NF-κB plays a pivotal role in regulating immune responses and inflammation through five genes belonging to NF-κB transcription factor family: NF-κB1 (p50/p105), NF-κB2 (p52/p100), RelA (p65), c-Rel and RelB which produce seven proteins acting as dimers and with a Rel Homology Domain in their sequence [32]. NF-κB controls the expression of more than 200 target genes involved in various biological processes such as cell proliferation, apoptosis, response to free radicals, and ultraviolet irradiation. 

A growing literature supports a major role for NF-κB in oncogenesis. In animal models, c-Rel homolog v-Rel induces avian lymphomas and leukemias being a highly oncogenic gene [33]. Furthermore, lymphomas, myelomas and leukemias may show genetic alterations of genes encoding for NF-κB subunits or other genes of the NF-κB signaling pathway leading to significant changes in NF-κB expression or activity [34,35,36]. High levels of c-Rel have been found in breast cancer where it has provided evidence for its function in carcinogenesis and in non-small cell lung carcinoma [37,38]. Moreover, RelA, RelB and NF-κB1 are constitutively activated in many human tumors. Increased nuclear translocation of RelA has been found in cervical and gastric carcinomas [39,40].

In about 20% HCC occurrences, as in other human cancers, inflammation seems to be the underlying pathology [41]. As matter of fact, a rich literature supports the role of the chronic liver inflammation induced by viral infection, hepatotoxic drugs, and metabolic injury in HCC development. However, the landscape of the hepatic burgeoning inflammation is very complex involving many different cell types including hepatocytes, hepatic macrophages i.e., Kupffer cells (KCs) and hepatic stellate cells (HSCs). Chronically injured hepatocytes activate inflammatory pathways releasing chemokines and cytokines, which in turn attract KCs and HSCs [42]. In this context, HSCs become activated thus playing a critical role in fibrogenesis which may drive towards HCC [43].

In animal models, liver injury has been linked to NF-κB activation with the response being increased in females and in mice fed a high-fat diet [41]. Moreover, KCs show NF-κB activation in response to damaged hepatocytes resulting in secretion of TNFα and IL-6 proinflammatory cytokines strongly involved in promoting fibrosis and HCC [44]. Indeed, through various inflammatory mediators and a tight interplay KCs and hepatocytes induce HSC activation. A growing literature points up a significant role of HSCs in hepatic immune response and inflammation mediated by NF-κB. In fact, p65 nuclear translocation is required for NO, TNF**α** and IL6 production in activated HSCs [45]. Of note, HSCs as well as hepatocytes and KCs also express TLR4—the most important LPS receptor—and activate NF-κB pathway following changes in gut microbiota, increased intestinal permeability and raised blood levels of LPS in mice fed a high-fat diet [46].

Although a moderate NF-κB hepatic activation represents a protective response against a persistent damage, its levels always increased may contribute to a chronic inflammatory state and to the development of hepatic insulin resistance [47].

However, it is not easy investigating NF-κB function in HCC pathogenesis and progression as NF-κB pathway inhibition may give apparently opposite effects in various animal models [48]. In this regard, conditional knockouts have been helpful showing that global deletion of IKK**β** (NF-κB activator) restrains HCC development; conversely, NF-κB liver deletion enhances susceptibility of liver damage and HCC [27]. 

**Figure 1:**
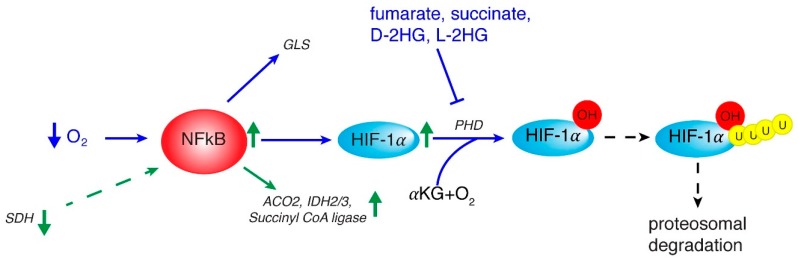
Interplay between NF-κB, HIF, and mitochondrial signals. The reported important role of NF-κB in tumor cell metabolism may originate from hypoxia that activates NF-κB which up-regulates *HIF-1α* gene. In turn NF-κB activates glutamine metabolism via glutaminase (GLS) and TCA cycle by *ACO2* (aconitase isoform 2), *IDH 2/3* (isocitrate dehydrogenase isoform 2 and 3) and *Succiyll CoA ligase* genes. HIF-1α stabilization takes place via L-2-HG, D-2-HG, succinate and fumarate which regulate PHD (oxygen-dependent prolyl hydroxylase) and subsequent HIF-1α proteosomal degradation. Events occurring in HCC are marked by green arrows.

Interestingly, NF-κB function as regulator of metabolism has been recently investigated. Among NF-κB subunits, RelA has been clearly reported to have an important role in tumor cell metabolism [49]. Of note, through IκBα degradation, hypoxia also activates NF-κB [50] which binds to HIF-1α gene promoter thus regulating its transcription (Figure 1) [51]. Through up-regulation of the metabolic SLC2A3 target gene, NF-κB may promote oncogenic transformation and enhance glycolysis [52]. Moreover, NF-κB regulates the transcription of aconitase 2, isocitrate dehydrogenase 3A (IDH3A), and succinyl-CoA ligase (SUCLA2) genes encoding three TCA cycle enzymes and IDH1 gene encoding a TCA-related protein in HCC cells (Figure 1) [53].

## 3. HIF in Regulating Metabolic Rewiring

Hypoxia inducible factors (HIFs) have a crucial role in adaptive response to hypoxia. HIFs transcription factors are made of an α subunit (oxygen-dependent) and a β subunit constitutively expressed—also known as aryl hydrocarbon receptor nuclear translocator (Arnt) [54]. The human genome contains three genes encoding α subunits (HIF-1α, HIF-2α, and HIF-3α) and three β subunits (Arnt1, Arnt2, and Arnt3). HIF-1α is the most expressed isoform and drives the acute response to hypoxia, while HIF-2α has a main role during chronic hypoxia. HIF-3α is less well characterized probably because of the presence of multiple HIF-3α variants [55]. In normally oxygenated tissues, α-subunits are rapidly degraded by oxygen-dependent prolyl hydroxylases (PHDs) and subsequent ubiquitination. PHDs are members of α-ketoglutarate-dependent dioxygenase family that function via oxidative decarboxylation of αKG in the presence of Fe^2+^ and O_2_ with hydroxylation of HIF-1α at two conserved proline residues with subsequent proteosomal degradation. When oxygen availability decreases, PHD inactivation leads to HIF-α stabilization and its nuclear import. Once in the nucleus, HIF-α dimerizes with a β subunit and activates the hypoxic response through transcriptional up-regulation of target genes [56]. Significantly, reduced concentration of oxygen in many human cancers [57]—compared to adjacent normal tissues—activates HIFα. Among HIFs, HIF-1 is the master regulator of the metabolic reprogramming occurring in cancer cells. HIF-1 promotes glucose catabolism through aerobic glycolysis and thus shifting glucose away from the TCA cycle by up-regulating the transcription of SLC2A1 and SLC2A3, encoding glucose transporters GLUT1 and GLUT3, respectively, hexokinase, the first enzyme of the glycolytic pathway, pyruvate kinase and lactate dehydrogenase A (LDHA) genes [58]. Furthermore, in the presence of low oxygen, HIF-1 transactivates PDK1 gene, encoding pyruvate dehydrogenase kinase 1, which phosphorylates and inhibits pyruvate dehydrogenase (PDH) complex thus reducing mitochondrial acetyl-CoA and increasing both pyruvate and lactate levels [59]. This metabolic condition induces HIF-1α gene expression suggesting the existence of a positive feedback loop supporting a cancerous metabolic phenotype where HIF-1 induction leads to PDK activity, elevated levels of pyruvate and lactate, and further increase in HIF-1 activity [60]. Increased levels of ROS also induce HIF-1α activation which in turn up-regulates its target genes fostering tumorigenesis.

Besides an oxygen-dependent mechanism, HIF-1 function is mediated by phosphatidylinositol 3-kinase and ERK mitogen activated protein kinase pathways, which promote cell growth and up-regulate HIF-1α translation [61].

High HIF-1α expression levels have been correlated with poor prognosis in patients with HCC [62]. Furthermore, increased HIF-1α activity in HCC samples has been associated with worse overall survival rates and lower response to external beam radiotherapy, thus suggesting HIF-1α as a predictive biomarker of treatment outcomes [63]. Of note, as for many cancers, there is a relationship between HIF-1α activity and resistance to drug-induced apoptosis of HCC cells [64]. Suppression of mitochondrial OXPHOS by up-regulation of glycolytic genes through HIF-1α was found to significantly correlate with a more aggressive HCC phenotype [65]. Intriguingly, HIF-1α activity is tightly controlled by TCA cycle signals since TCA cycle intermediates such as succinate, fumarate and αKG as well as the related oncometabolite L-2 hydroxyglutarate (L-2-HG) regulate HIF-1α proteosomal degradation (Figure 1). These findings highlight new features of HIF-1α function in metabolic reprogramming and suggest a complex interplay between HIF-1α and TCA cycle rewiring in cancers, including HCC.

## 4. TCA Cycle and Related Enzymes in HCC

Several nuclear genes encode enzymes catalyzing TCA cycle biochemical reactions. It is well-known that through a series of cyclic reactions, TCA cycle is involved not only in energy production and electron transfer processes, but also in synthesis of intermediates which are used as building blocks for macromolecules. Fuel feeding of TCA cycle differs in normal and cancer cells. A growing body of evidence indicates a widespread dysregulation of TCA cycle and related enzymes either at expression or activity level in cancers such as HCC frequently related to cell transformation and progression.

### 4.1. Citrate Synthase

The human gene encoding citrate synthase (CS) maps to chromosome 12 (12q13.3). CS localizes in the mitochondrial matrix and catalyzes the synthesis of citrate from acetyl-CoA and oxaloacetate (OAA), thus regenerating CoA. This is the first step and the rate-limiting reaction of the TCA cycle [66]. CS presents either up-regulation or increased enzymatic activity in various cancer types such as ovarian and pancreatic cancer [67,68]. Noteworthy, CS enzyme activity has been reported to be increased in HCC [69]. More recently, RNAseq analysis—then verified in multiple microarray data sets—identified CS overexpression among unique 22-carbon-metabolism-gene signature of HCC [70]. Furthermore, Zhang et al. have also demonstrated that CS knockdown strongly decreases HCC cell proliferation and hepatospheroid formation in low-glucose condition when compared to respective controls. Since HCC is associated with a metabolic reprogramming toward raised glycolysis and lipogenesis, increased CS gene expression and activity can provide cytosolic substrate for membrane lipid synthesis. According to the metabolic function of CS in HCC, Gao et al. found increased levels of citrate in HCC samples than healthy controls [71]. These data point out CS gene silencing or inhibition as a potential way to partially reduce the malignant phenotype of HCC.

### 4.2. Isocitrate Dehydrogenase

**Figure 2:**
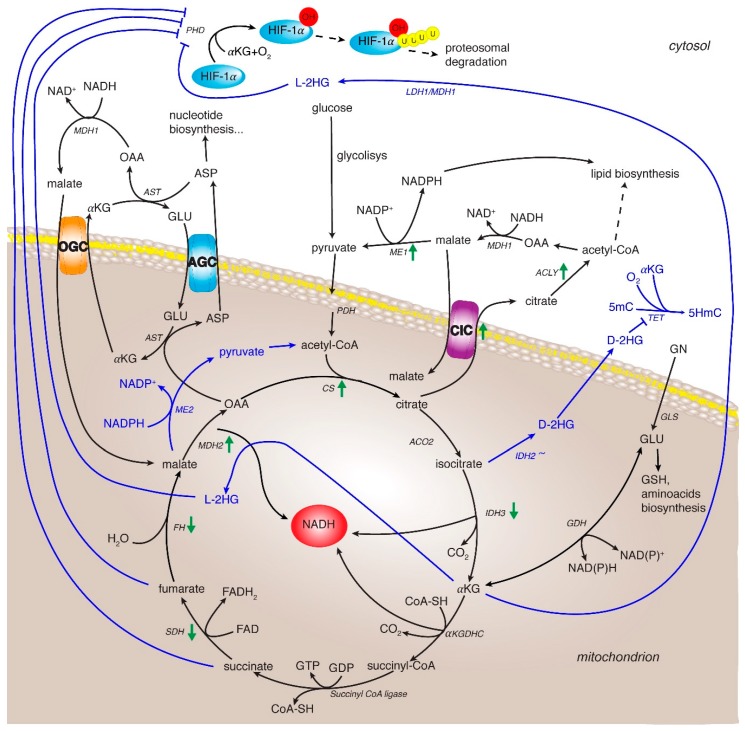
TCA cycle rewiring and mitochondrial signaling in cancer and HCC. The TCA cycle metabolic flux is represented by black arrows and specific enzymes. Three of them catalyze irreversible reactions: the citrate synthase, the isocitrate dehydrogenase 3 and the α-ketoglutarate dehydrogenase complex. Blue lines indicate the reprogramming of metabolic reactions in cancer. Green arrows indicate up- and down-regulation of expression and/or activity of TCA cycle and related enzymes in HCC. Enzyme abbreviations: CS: citrate synthase; ACO2: aconitase isoform 2; IDH2/3: isocitrate dehydrogenase isoforms 2 and 3; αKGDHC: αKG dehydrogenase complex; SDH: succinate dehydrogenase; FH: fumarate hydratase; MDH1 and 2: malate dehydrogease isoform 1 and 2; PDH: pyruvate dehydrogenase; GLS: glutaminase, GDH: glutamate dehydrogenase; CIC: citrate carrier; AGC: aspartate/glutamate carrier; OGC: oxoglutarate carrier; ACLY: ATP-citrate lyase; ME1 and 2: malic enzyme isoform 1 and 2; AST: aspartate aminotransferase; PHD: oxygen-dependent prolyl hydroxylase; TET: Ten-eleven translocation family protein. Metabolite abbreviations: 2-L-HG (2-L-hydroxyglutarate) and D-2-HG (2-D-hydroxyglutarate), 5-mC: 5-methylcitosine; 5HmC: 5-hydroxymethylcytosine; OAA: oxaloacetate; αKG: α-ketoglutarate; ASP: aspartate; GLU: glutamate.

The isocitrate dehydrogenase (IDH) family consists of three isoforms (IDH1, IDH2, and IDH3) responsible for the decarboxylation of isocitrate to αKG. The cytosolic and peroxisomal IDH1, encoded by IDH1 gene on chromosome 2, catalyzes both the oxidative decarboxylation of isocitrate to αKG thus reducing NADP^+^ to NADPH and the reductive carboxylation of αKG—that is the reverse reaction—to isocitrate with concomitant oxidation of NADPH to NADP^+^. IDH2 mitochondrial isoform is encoded by IDH2 gene mapping to chromosome 15q26.1 and catalyzes the same reversible reaction as described for IDH1 [72]. IDH1 and IDH2 isoforms work as homodimers [73] while IDH3 is an heterotetramer made up of two alpha subunits (IDH3A), one beta subunit (IDH3B), and one gamma subunit (IDH3G) [74]. Unlike IDH1 and IDH2, IDH3 has a well-established role in TCA cycle catalyzing the irreversible conversion of isocitrate to αKG while reducing NAD^+^ to NADH. IDH1 and/or IDH2 mutations occur in glioma, and rarely in other cancers [75,76]. Of note, a just published work—by re-clustering and analyzing a genomic datasets including 196 patients from The Cancer Genome Atlas group—identified a more aggressive subset of HCC patients showing IDH1/2 mutations status and a worse survival than the other subsets [77]. These mutations result in the gained function producing the oncometabolite D-2-hydroxyglutarate (D-2-HG), which induces decreased levels of 5-hydroxymethylcytosine (5-HmC) by inhibiting Ten-eleven translocation (TET) family of 5-methylcitosine (5-mC) hydroxylases, an αKG dependent dioxygenase (Figure 2) [78]. Furthermore, IDH3A has been found up-regulated in HCC cell lines treated with a combination of celecoxib and sorafenib, two drugs showing synergistic anti-proliferative and pro-apoptotic effects on HCC cells [79]. The metabolic impact of IDH1/2 mutations as well as IDH3A down-regulation in HCC could be linked to a reduced NADPH production, which results insufficient to maintain a favorable GSH:GSSG ratio thus increasing steady-state ROS and in turn oxidation of cellular components and DNA mutation rate, as already reported for others cancers involving IDH1/2 mutations [80,81].

### 4.3. α-Ketoglutarate Dehydrogenase Complex

The α-ketoglutarate dehydrogenase complex (KGDHC) is a TCA cycle enzymatic complex that catalyzes the synthesis of succinyl-CoA via decarboxylation of αKG. This complex is formed from three proteins present in multiple copies: α-ketoglutarate dehydrogenase (E1k subunit, EC 1.2.4.2), dihydrolipoamide succinyltransferase (E2k subunit, EC 2.3.1.12), and dihydrolipoamide dehydrogenase (E3 subunit, EC 1.6.4.3), and requires five cofactors: thiamine pyrophosphate, lipoic acid, coenzyme A, FAD, and NAD^+^. In the reaction catalyzed from KGDHC, E1k transfers two electrons from αKG to the lipoyl group which is bound to E2k subunit. E3 subunit catalyzes the oxidation of reduced lipoyl group of E2k transferring the pair of electrons to the prosthetic group FAD and finally to NAD^+^, forming NADH. The E1k and E3 subunits are noncovalently bound to a core formed by E2k subunits [82,83]. KGDHC is activated by low concentrations of Ca^2+^ (10^−7^−10^−5^ M) and ADP (∼10^−4^ M for half-maximum activation) [84,85], and it is inhibited by high NADH and its own product, succinyl-CoA [86,87,88].

Recently, a KGDHC alteration was related to HIF-1α stabilization [89]. In normoxia HIF-1α levels are maintained very low due to proteosomal degradation after hydroxylation by PHDs, α-ketoglutarate dependent dioxygenases. Interestingly, the disruption or inhibition of KGDHC function may cause a conversion of αKG to the oncometabolite L-2 hydroxyglutarate (L-2-HG) via spurious activity of LDHA and MDH1 in the cytosol, and via MDH2 in the mitochondria (Figure 2) [90]. Consequently, NADH accumulation and chain respiratory alteration facilitate this conversion. L-2-HG was the predominant enantiomer synthesized in these conditions and is the only compound able to inhibit PHDs with a stabilization of HIF-1α [89]. Thus, αKG-dependent PHDs would be regulated by L-2-HG/αKG ratio as αKG supplementation or KGDHC inhibition leads to a low increase of L-2-HG levels in normoxia condition, and high increase in hypoxia condition [91]. In this context, αKG accumulation promotes the formation of L-2-HG thus regulating PDH function and in turn HIF-1α stability.

Of note, KGDHC has been also found in the nucleus of human cell lines bound to lysine acetyltransferase 2A (KAT2A, also known as GCN5) in the promoter regions of genes and the resulting complex acts as a histone H3 succinyltransferase. When α-KGDH complex does not enter the nucleus, or KAT2A gene expression is reduced, inhibition of tumor cell proliferation and tumor growth was observed [92].

### 4.4. Succinate Dehydrogenase

Succinate dehydrogenase (SDH) complex, localized in the inner mitochondrial membrane, is composed of four nuclear-encoded subunits (SDHA, SDHB, SDHC, and SDHD). This highly conserved heterotetrameric enzyme catalyzes the oxidation of succinate to fumarate while simultaneously reducing FAD to FADH_2_ with subsequent transfer of electrons to ubiquinone. Known as mitochondrial respiratory chain complex II, SDH is the only complex which takes part in both TCA cycle and the electron transport chain and unlike most of the TCA cycle enzymes, does not show a cytosolic counterpart. Moreover, SDH is the only respiratory chain complex consisting of subunits totally encoded by nuclear genes mapping at chromosome bands 5p15 (SDHA), 1p36 (SDHB), 1q21 (SDHC), 11q23 (SDHD), 19q13 (SDHAF1), and 11q12 (SDHAF2), respectively [93]. Mutations in genes encoding different subunits of SDH have been associated with succinate accumulation and are causative of hereditary paraganglioma and pheochromocytoma [94]. The germline heterozygous mutations predisposing to hereditary paraganglioma and pheochromocytoma cause inactivation of the protein function. However, the neoplastic transformation takes place when there is the loss of the remaining wild type allele in the somatic cells (loss of heterozygosity) inducing the complete loss of SDH function. Thus, SDH is classified as a classical tumor suppressor gene [95].

Mutations in SDH genes were identified in several other cancers including thyroid, gastrointestinal stromal, neuroblastoma, renal and ovarian cancer [96]. SDHA and SDHB genes have also been found down-regulated in HCC [97]. Additionally, SDHB low expression levels in patients with HCC induced a metabolic shift from aerobic respiration to glycolysis and were associated with advanced tumor stage and poor survival rate [98].

Very recently, Li et al., investigating the expression levels of the genes encoding SDH subunits found a significant down-regulation of all four subunits which results in reduced SDH activity and increased ROS and succinate levels in HCC. Moreover, deficient SDHC activity promotes HCC cell growth and migration as well as NF-κB signaling [99].

The down-regulation of one or more SDH subunits associated with a decrease of SDH activity in HCC—reported from a growing literature in recent years—explains the accumulation of succinate also observed in HCC [71]. Increased levels of succinate activate HIF-1 which in turn up-regulates metabolic genes switching to the Warburg effect [26].

Interestingly, SDH activity is also impaired in injured hepatocytes thus inducing increased intrahepatic levels of succinate. The latter has been reported to activate HSCs through the membrane receptor GPR91 pointing out the succinate as a signal metabolite in hepatic fibrosis [100]. These recent findings clearly indicate a critical tumor-suppressive function for SDH and highlight its crucial in HCC pathogenesis and metabolic reprogramming.

### 4.5. Fumarate Hydratase

Fumarate hydratase (FH) is encoded by FH gene and exists in cytosolic and mitochondrial forms, differing only in the translation start site used. The mitochondrial enzyme derives from the N-terminal extended form that contain the FH mitochondrial targeting sequence, the removal of which results in the same form as in the cytoplasm. FH catalyzes the reversible conversion of fumarate to L-malate [101]. The mitochondrial form participates in the TCA cycle, whereas the cytosolic FH is involved in different pathways including the urea and the purine nucleotide cycles [102].

FH has been proposed to function as a tumor suppressor. In fact, deficiency of FH induces accumulation of the mitochondrial fumarate which promotes HIF-1α stabilization by inhibition of the prolyl hydroxylases (PHDs) [103].

PHD inhibition, leading to high levels of HIF1, and consequent activation of oncogenic targets essential for tumorigenesis, increases angiogenesis and glucose metabolism [104].

Interestingly, FH as well as SDH genetic mutations are associated with a methylator phenotype affecting particularly DNA methylation of promoter regions typically with a transcriptional down-regulation of tumor suppressor genes [105]. DNA and histone methylation increase reflects DNA and histone demethylase alterations. Therefore, FH mutations lead to fumarate accumulation and consequently to TET inhibition resulting in a rise of 5-mC/5-HmC ratio. In paraganglioma, this increase is related to a metastasis progression and invasiveness [106], and the DNA CpG island hypermethylation has been associated with a poor prognosis [107]. Therefore, FH deficiency may lead to epigenetic reprogramming in different cancers, as gastrointestinal stromal tumors and renal cancer, characterized by FH mutations.

Moreover, FH is also involved in the response to the nuclear DNA double strand breaks [108] to maintain genomic stability [109]. Consequently, FH inactivation takes part in cancer development by inducing a loss of genome integrity.

Interestingly, in HCC cells with portal vein thrombosis, FH expression was found more than 2-fold decreased and this down-regulation leads HCC cells to become aggressive and able to invade the vessels [110].

### 4.6. Malate Dehydrogenase

Malate dehydrogenase (MDH) is encoded by two different genes: MDH1, encoding the cytosolic enzyme and MDH2 gene encoding the mitochondrial enzyme. Both enzymes are part of the MAS and catalyze the reversible reaction of OAA to malate in the presence of NADH. MDH1 is involved in the oxidation of NADH to NAD^+^, which is then used for continuing the glycolysis. MDH2, besides being part of TCA cycle, converts the cytosolic malate—produced by MDH1 and transported across the mitochondrial inner membrane—to OAA with reduction of NAD^+^ to NADH, which is used to mitochondrial electron transport chain.

MDHs are stable as dimers and each subunit contains two domains: the NAD-binding domain, in the amino-terminal half, and the carboxy-terminal domain characterized by amino acids necessary for catalysis and substrate binding site. Furthermore, MDH is allosterically regulated by different metabolites. In particular, MDH2 is activated by elevated concentrations of malate, whereas high levels of OAA inhibit this reaction [111,112]. An interesting effect is shown by citrate that functions as an inhibitor of reduction of OAA in malate, but activates MDH2 when malate and NAD^+^ levels are very high making the enzyme adaptable to metabolic changes [113]. The importance of citrate regulation is also corroborated by the existence of a complex between MDH2 and CS whose kinetic advantage is to channel OAA from MDH2 to CS. In fact, the MDH2 reaction is favorite in direction of reduction of OAA, but the formation of enzyme complex drives OAA to synthesize the citrate [114].

In cancer cells the NAD^+^/NADH homeostasis is maintained by both glycolysis and oxidative phosphorylation. In this scenario, MDH—as part of MAS—represents an important link between these two processes, consequently, MDH induced expression was investigated as malignancy marker [115].

Recently, MDH2 was associated with several type of cancer in which MDH2 expression levels were found increased. Indeed, the regulation of MDH2 activity was proposed as target to mediate resistance cancer cells to chemotherapy [116]. Accordingly, MDH2 inhibition by different compounds causes a decrease of mitochondrial respiration by the reduction of NADH levels and mitochondrial oxygen consumption with an increase of oxygen content which can lead to HIF-1 degradation [117]. Of note, two Oncomine datasets (https://www.oncomine.org/) report MDH2 up-regulation in HCC samples highlighting its role in tumorigenic metabolic reprogramming of liver cells.

### 4.7. Citrate/Pyruvate Shuttle 

TCA cycle-derived citrate is exported from mitochondria to the cytosol, through CIC, a member of the mitochondrial carrier family encoded by *SLC25A1* gene [22]. In the cytosol, ACLY catalyzes the conversion of citrate and CoA into OAA and acetyl-CoA. The latter is used for fatty acids and sterol biosynthesis, whereas MDH1 reduces OAA to malate, which is then converted to pyruvate via malic enzyme 1 (ME1) thus producing cytosolic NADPH and H^+^ (necessary for fatty acid and sterol synthesis) [118]. First, through the mitochondrial citrate export, citrate/pyruvate shuttle replenishes cytosolic substrates for lipogenesis, very raised in tumors. Thus, CIC plays a key role in regulating the amount of citrate transported. For this reason, *SLC25A1* gene is tightly regulated by transcriptional factors and epigenetics in physiological and pathological conditions [119,120,121]. Of note, both *SLC25A1* and *ACLY* genes are up-regulated via NF-κB in LPS and TNFα-activated M1 macrophages [122,123,124]. Therefore, it could be likely hypothesized a role for NF-κB in *SLC25A1* and *ACLY* up-regulation found in a variety of cancers, including HCC.

Other effects of the citrate/pyruvate shuttle are NADH oxidation—useful for the increased aerobic glycolysis of tumor cells—and NADPH production. Cancer cells can require more NADPH for lipid metabolism, redox homeostasis and molecular biosynthesis than their normal counterparts [125]. By reducing NADP^+^ to NADPH, ME1 has been reported to be a major metabolic enzyme which contributes to the balance of intracellular NADPH for macromolecular biosynthesis and protects from excessive oxidative stress [126].

Indeed, immoderate levels of ROS often found in cancer cells, may damage cellular macromolecules and lead to apoptosis [127]. In this context, citrate/pyruvate shuttle through ME1 enhances NADPH production preserving redox homeostasis (Figure 2). Accordingly, ME1 gene silencing in HCC cell lines induces increased levels of ROS and reduced NADPH [128].

In gastric cancer, ME1 has also a key function in providing NADPH for glutathione and ROS homeostasis, which was critical for cancer cell survival under energy stress conditions, such as glucose limitations. Furthermore, aberrant ME1 gene expression was associated with poor prognosis of gastric cancer. Exploring the therapeutic potential of ME1 in cell line–based as well as patient derived xenograft models, gene silencing of ME1 significantly suppressed tumor growth increasing cell apoptosis [129]. ME1-repressed cells show a decreased tolerance to low-glucose condition and diminished migration and invasion of nasopharyngeal cancer cells [130]. ME1 overexpression also confers radiation resistance in lung cancer [131].

About further implications in HCC progression, a recent study reports an ME1 up-regulation in 65 HCC when compared to peritumoral tissues. ME1 gene silencing inhibits migratory and invasive properties of HCC cells. Of note, ME1 overexpression was significantly associated with reduced overall survival and reduced progression-free survival of HCC patients [128].

### 4.8. The Malate/Aspartate Shuttle (MAS)

The maintenance of the REDOX state of cancer cell is crucial for promoting tumor survival and proliferation [132]. In recent years, a large amount of evidence has highlighted the importance of redox homeostasis in cancer cells, including HCC.

A central role in the redox homeostasis is played by the MAS, in which two mitochondrial carriers, the aspartate/glutamate carrier isoform 1 (AGC1), encoded by the *SLC25A12* gene and the glutamate/aspartate isoform 2 (AGC2) encoded by the *SLC25A13* gene, are involved [133]. They are differently expressed in various tissues: AGC1 is abundant in brain, pancreas, skeletal muscle, but at a very low level in liver, whereas AGC2 is ubiquitous and particularly abundant in liver and kidney [134]. Despite the evidence that the MAS activity increased in hepatoma and leukemia since 1976 [135], only recently the functional importance of the transported metabolites (glutamate and aspartate) and related mitochondrial carriers in HCC has been studied.

AGC1 isoform is significantly upregulated in HCC, whereas AGC2 is downregulated with respect to normal liver cells. FOXA2, a well-known tumor suppressor and a transcriptional regulator of *SLC25A13* gene, can be responsible for its high expression levels in liver and its downregulation in HCC [136].

Concerning *SLC25A12 gene*, an in-depth analysis by using Oncomine and Human Proteome Atlas datasets also showed a strong upregulation in other types of tumors. Epigenetic mechanisms promote *SLC25A12* up-regulation in HCC as its expression levels are depend on acetyl-CoA amount. In fact, acetylation of H3 and H4 histones as well as CREB binding at *SLC25A12* promoter are reduced during ACLY inhibition. Of note, *SLC25A12* but not *SLC25A13* gene silencing induces a cell cycle arrest in G1 phase of HCC cells [137]. Likely, an epigenetic reprogramming including *SLC25A12* control occurs to enable liver cancer cell survival and growth [138,139]. AGC1 plays a dual role: to maintain NADH/NAD^+^ homeostasis, by providing NADH to the mitochondria, and to support aspartate needed for the increased metabolic biosynthesis, in particular for the nucleotide synthesis [140,141]. In fact, aspartate as well as deoxynucleotide treatment reverses cell proliferation of *SLC25A12*—but not *SLC25A13*-silenced HCC cell lines.

Mitochondrial aspartate export is essential for de novo nucleotide synthesis because of the low aspartate cell permeability [23]. Of note, metabolic and epigenetic reprogramming as well as genetic mutations can compromise the mitochondrial membrane potential of tumor cells [142,143], required for aspartate biosynthesis [144].

Thus, the increased activity of AGC1 is also essential for redox homeostasis by moving NADH to mitochondria, promoting mitochondria energization and in turn mitochondrial aspartate export of HCC cells. A specific function for AGC1 in redox balance of cancer cells has been also pointed up by Amodeo et al. [145]. Therefore, AGC1 function ensures enough aspartate for both redox homeostasis and cell proliferation. Kinetic evidence is in support of this hypothesis. In fact, recombinant AGC1 and AGC2 reconstituted in proteoliposomes show an identical half-saturation constants (Km), but a different Vmax (much lower for AGC1 than that of AGC2) [146].

Hence, although both AGC1 and AGC2 are involved in MAS, AGC1 has a specific role in nucleotide biosynthesis of HCC cells. Accordingly, aspartate derived from several metabolic pathways has been recently identified as a critical intermediate for de novo nucleotide biosynthesis of dividing cells [140]. Of note, aspartate drives cell proliferation in citrullinemia type I (with arginosuccinate synthase deficiency) as well as in tumors showing arginosuccinate synthase down-regulation [147].

The role of AGC1 as cytosolic aspartate supplier was further investigated by Furkan Alkan which demonstrated that AGC1 knockdown slows in vivo growth of mouse lung and pancreas cancer cells, reduces cellular NAD^+^/NADH ratio, and impairs aspartate supply to cytosol. Tumor growth was further reduced when in vivo cells were treated with CB-839, a GLS inhibitor, suggesting that loss of AGC1 can synergize with GLS inhibitors. Proliferation was restored upon exogenous aspartate supplementation even when aspartate aminotransferase was blocked suggesting that aspartate is not required to replenish TCA cycle for promoting cell proliferation [148].

When compared to AGC proteins, MDH1 and oxoglutarate mitochondrial carrier (OGC) gene expression—other components of MAS—seem less regulated in cancer cells. Indeed, their expression levels are not chancing in many cancer datasets analyzed (https://www.oncomine.org/), including HCC. Intriguingly, the critical role played by MAS in HCC is ascribable, at least in part, to AGC proteins whose genes are tightly regulated by different molecular mechanisms during liver cell tumorigenesis.

### 4.9. Glutamine Metabolism

Glutamine metabolism is also important in the maintenance of redox state. It is critical during cell growth and proliferation since it can act as precursor in the biosynthesis of different amino acids, proteins, nucleotides, and other molecules. Recently, glutamine has gained attention because its metabolism rapidly changes in cancer cells. Notably, glutamine deprivation is an effective way to suppress cancer cell growth [149].

Through different transport systems for amino acids [150] glutamine cross the plasma membrane. Then, by a still unknown mitochondrial carrier, enters mitochondria where it can be metabolized by an oxidative and/or reductive metabolism [151]. In the presence of oxygen and active respiration, GLS and GDH transform glutamine in αKG, which enters TCA cycle, supporting energy production, and some substrates for other biosynthetic pathways. In the hypoxic conditions αKG from glutamine may be converted into citrate that is exported from mitochondria into cytosol.

Glutamine also supports the production of glutathione (GSH), the most abundant antioxidant in hepatocytes that helps to prevent oxidative stress in most cells and decreases ROS levels [152]. Through glutaminolysis, glutamine is converted into glutamate, one of the glutathione precursors. In the antioxidant function, the reduced GSH is oxidized to the dimer form GSSG. The GSH/GSSG ratio is a good indicator for redox balance. The regeneration of GSH requires the glutathione reductase enzyme and reducing equivalents in the form of NADPH.

HIF-1 contributes to the tight regulation of glutamine metabolism occurring in cancer cells. This control seems to be one of the causes of carcinogenesis [153]. In addition, GLS up-regulation in cancer is mediated by NF-κB [154].

Concerning HCC, the role of glutamine is not yet clear, because conflicting data have been reported so far. De Waal et al. [155] reported that glutamine is metabolized via oxidative way in HCC whereas Bjornson et al. [156] showed that the transformation of glutamine to αKG is down-regulated in HCC with respect to the normal cells. Interestingly, Yuneva et al. [13] reported that glutamine metabolism could depend on initiating liver lesion in HCC. Likely, this is due not only to microenvironmental conditions, but also to genetic gene alterations.

## 5. TCA Cycle and Related Enzymes as New Potential Therapeutic Targets

### 5.1. Targeting MDH2 and Malic Enzymes

A first potential anticancer target protein of TCA cycle is MDH2, the mitochondrial isoform of malate dehydrogenase. In prostate cancer cells, MDH2 overexpression is associated with a shorter survival and a chemoresistance. On the contrary, MDH2 knockdown via shRNA leads to cell proliferation decrease and a chemosensitivity increase [116].

**Figure 3:**
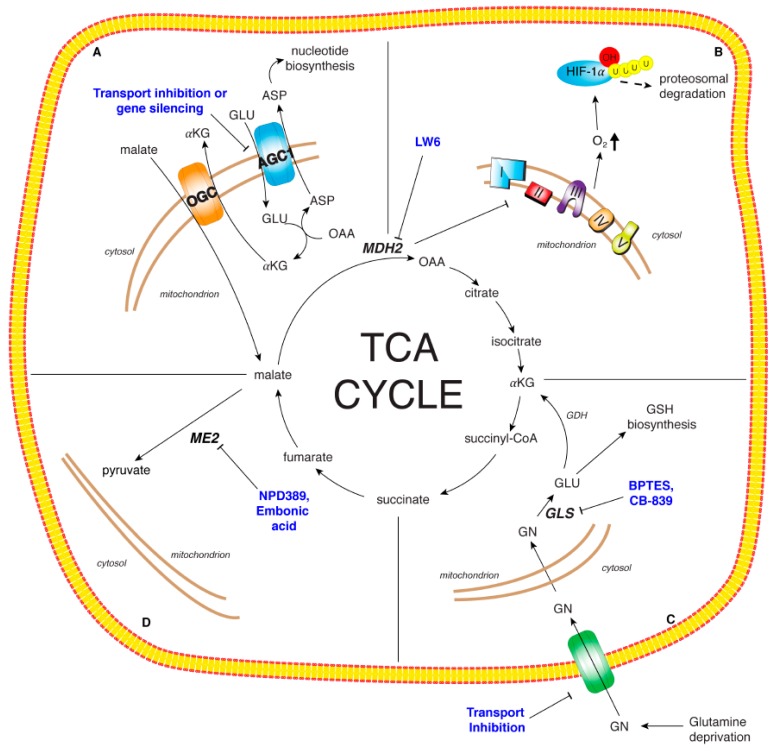
Therapeutic targets of TCA cycle and related enzymes. Panel A: The malate/aspartate shuttle essential for the maintenance of the REDOX state of cancer cell. The specific up-regulation of AGC1 in HCC may by targeted through gene silencing or specific protein transport inhibitors. Panel B: Malate dehydrogenase isoform 2 (MDH2): LW6, an aryloxyacetylamino benzoic acid analog, was identified as MDH2 inhibitor leading to TCA cycle impairment, mitochondrial respiration decrease and oxygen concentration increase which in turn induces HIF-1α degradation. Panel C: Glutamine metabolism critical for cancer cell growth and proliferation. The modulation of glutamine metabolism has been proposed via glutamine deprivation, transport inhibition, reduction of glutamine metabolic enzyme activities. BPTES and CB-839 represent two promising inhibitors of GLS. Panel D: Malic enzyme isoform 2 (ME2): NPD389 and embonic acid are non-competitive inhibitors of ME2. ME2 inhibition could impair cancer cell proliferation and growth.

MDH2 was identified as target of LW6, an aryloxyacetylamino benzoic acid analog, potently able to inhibit HIF-1α accumulation. Indeed, MDH2 inhibition through LW6 leads to TCA cycle and mitochondrial respiration impairment thus increasing oxygen concentration and in turn HIF-1α degradation (Figure 3) [117]. These data underline the potential role of MDH2 inhibition in cancer therapeutics and open the way to new inhibitors.

Related to MDH2 inhibition and mitochondrial malate accumulation, another potential anticancer target is malic enzyme isoform 2 (ME2). Three isoforms of malic enzyme have been identified: the cytosolic NADP^+^-dependent isoform ME1, the mitochondrial NAD(P)^+^-dependent isoform ME2 and the mitochondrial NADP^+^-dependent isoform ME3. MEs catalyze oxidative decarboxylation of malate to pyruvate and CO_2_, with the production of NADH or NADPH [157]. ME2 is active in the presence of high levels of amino acids and provides mitochondrial pyruvate thus increasing TCA cycle flux in limiting glucose condition [158].

ME2 is considered a potential anticancer target considering his increased activity in cells as Morris hepatomas [159]. Furthermore, it was demonstrated that *ME2* knockdown impairs the proliferation and the growth of different cancer cell lines. No inhibitors of human ME2 are currently known, but When et al. have identified, through a high-throughput screening system, NPD389 as a potent non-competitive inhibitor for ME2 [160]. However, the cytotoxic effect of this compound in different cancer cell lines was still not investigated. In vitro ME2 activity inhibition was also investigated with the natural compound embonic acid which also shows a non-competitive inhibition against ME2 and a reduction of growth of H1299 cancer cells (Figure 3). The cytotoxic activity of embonic acid has been evaluated, but the direct involvement of ME2 must still be investigated.

Furthermore, ME1 is used as prognostic and predictive marker for radiotherapy in cancer suggesting a critical role in metabolic reprogramming of cancer cells [128]. Although no ME1 inhibitors are currently being evaluated in clinical trials, a strategy combining ME1 and glycolysis inhibition, would provide an effective therapeutic option for cancer treatment. Since ME1 overexpression is critical for HCC redox homeostasis and is significantly associated with reduced overall survival of HCC patients, ME1 inhibition could be promising even in this malignancy.

### 5.2. Targeting MAS

The specific up-regulation of AGC1 in HCC is a very interesting new finding to address new therapeutic targets. Its knockdown strongly decreases HCC cell growth and migration by reducing cytosolic aspartate levels for nucleotide biosynthesis (Figure 3). Thus, inhibiting AGC1 expression leads to a significant reduction of aspartate supply to the cytosol. Different strategies may be employed to reduce AGC1 expression.

Since its overexpression is due to epigenetic regulation, using epigenetic modifier could be tested. It is widely demonstrated that epigenetic alterations are associated with different mechanisms of proliferation and metastasis in several types of cancer, including HCC [139,161,162,163]. Histone acetylation is one of most important epigenetic mechanisms, regulating cellular events such as differentiation, proliferation, and cell cycle. Deregulated histone acetyltransferases and histone deacetyltransferase activity plays a role in the development of a range of cancers. Of note, histone acetylase coactivator p300 contributes to the development of hepatic steatosis via histone and non-histone acetylation [164]. It has also been found p300 high expression in HCC. In addition, p300 overexpression is a biomarker for poor prognosis of HCC patients [165]. Consequently, inhibitors of these epigenetic enzymes have potential as anticancer agents.

Beyond repression at transcriptional level, inhibition of transport activity could be another strategy (Figure 3). Currently, specific AGC1 inhibitors are not available; however, they could be found among the best binders of a virtual screening as well as among those small molecules in the ligand-clusters containing the AGC reference substrates and the traditional mitochondrial carrier inhibitors [146,166].

### 5.3. Targeting Glutamine

In recent years glutamine metabolism has become a very interesting topic target in cancer therapy. Glutamine metabolism can be modulated through deprivation, uptake restriction, usage of glutamine analogs, glutaminolysis inhibition, and metabolic regulators of cancer cells. Although these strategies have been tested in different types of cancer other than HCC, their efficacy in this malignancy is not excluded.

There are multiple effects of glutamine deprivation in cancers including apoptosis and autophagy induction, increase of mitochondrial ROS, decrease of GSH levels [167], and cell cycle arrest [168].

Another strategy to affect glutamine metabolism concerns the transport inhibition (Figure 3). Some transport systems for amino acids to cross plasma membrane, such as Na^+^-dependent system A, Na^+^-dependent system N, Na^+^-dependent system ASC (alanine serine cystein) and Na^+^-independent system L are used for glutamine transport. Two of them, ASCT2 and LAT1 up-regulated in cancer [150], have been tested for glutamine transport inhibition. ASCT2 inhibition decreases glutamine uptake and cell cycle progression [169]. Inhibition of LAT1 leads to a reduction of cell proliferation and viability of different cancer cells [170]. Furthermore, another target can be the liver receptor homolog (LRH-1), implicated in coordinating glutamine-induced metabolism and hepatocarcinogenesis. Xu et al. demonstrated that LRH-1 deletion inhibited HCC cell proliferation through different mechanism, such as reduction of glutamine deamination and glutaminolysis and inhibition of mTOR signaling [171].

The most promising therapeutic target so far is the inhibition of GLS by the allosteric GLS-selective inhibitor, bis-2-(5-phenylacetamido-1,3,4-thiadiazol-2-yl)ethyl sulfide (BPTES) and BPTES like-drug CB-839 (Figure 3). BPTES shows an antitumor effect in different tumor types, and a prolonged survival in a model of murine hepatocellular carcinoma without toxic side effects [154]. Considering the moderate effect together with the poor metabolic stability and the low solubility, a new potent allosteric inhibitor of GLS, CB-839, was clinically tested. Moreover, CB-839 affects TCA cycle reprogramming, glutathione production and amino acids synthesis [172]. Thus, this compound is in Phase I clinical trials for treatment of hematological and solid tumors (NCT02071927 and NCT02071888). Considering previous findings, it could also be supposed to have beneficial effects in HCC.

## 6. Conclusions

Metabolic pathway alterations characterize HCC resulting in a global metabolic reprogramming. Among them, significant changes in TCA cycle and related enzymes correlate with cancer cell transformation and progression. The maintenance of the REDOX state is also crucial for promoting tumor survival and proliferation. In this review, following an updated analysis of TCA cycle, related shuttles, and glutamine fate in HCC, we propose that targeting these pathways might have significant clinical implications and open new windows in the therapeutic strategies. However, incoming studies need to deepen our understanding of TCA cycle rewiring in HCC and better develop therapeutic tools for this malignancy.
